# Successful pregnancies and a live birth after intracytoplasmic sperm injection in globozoospermia

**DOI:** 10.4103/0974-1208.57228

**Published:** 2009

**Authors:** Manish R Banker, Pravin M Patel, Bharat V Joshi, Preeti B Shah, Rakhi Goyal

**Affiliations:** Pulse Women's Hospital, Ahmedabad, India

**Keywords:** Globozoospermia, intracytoplasmic sperm injection, male factor infertility

## Abstract

Globozoospermia is a severe form of teratozoospermia characterized by round-headed acrosomeless spermatozoa. Here we present two successful pregnancies and a live birth after intracytoplasmic sperm injection.

## INTRODUCTION

Globozoospermia is a rare disorder causing male infertility (incidence <0.1%).[1] It is diagnosed by the presence of 100% round headed spermatozoa and was first described by Schirren *et al*.[2] Many case reports have elucidated the morphological and functional abnormalities in cases of globozoospermia but the underlying causes remain unclear.[2,3]

The main morphological defects in the disorder include an absent or severely malformed acrosome.[3] An increased aneuploidy rate has been observed in some cases, on cytogenetic analysis, mostly in the acrocentric (13, 14, 15, 18 and 21) and sex chromosomes but the findings are similar to other types of male infertility.[4] Increased DNA fragmentation and DNA damage has also been noted.[5,6] The pathogenesis of globozoospermia most probably originates in spermiogenesis, especially in acrosome formation and sperm head elongation.[1–3]

In globozoospermia there is an absence of acrosomal structures leading to absence of spermatozoa binding to zone pellucida.[7] Before the advent of ICSI, these men were considered sterile as conventional *in vitro* fertilization (IVF)[8] and sub zonal insemination,[9] failed in these patients. However, even with use of ICSI, variable fertilization and cleavage rates have been described in these cases, with some reporting fertilization failure due to failure of oocyte activation.[10] Various pregnancies and live births reported till now are either with conventional ICSI or with use of oocyte activation measures like calcium ionophore.[11] Here we report two successful cases of pregnancies in couples with globozoospermia with use of ICSI without oocyte activation.

## CASE REPORTS

### Case 1

The couple presented to us with a primary infertility of six years duration. The 24-year-old woman had regular ovulatory cycles, normal hormonal profile and normal laparoscopy and hysteroscopy (carried out earlier). The previous spermiogram report of her 29-year-old husband showed a sperm count of 48 million/ml, with 30% actively motile and 36% morphologically abnormal sperm. They had undergone three intra uterine insemination cycles previously. Two fresh semen analyses at our clinic showed normal count and motility and 100% globozoospermia. The woman was stimulated using long protocol with 0.5 mg of Leuprolide acetate (Lupride^®^ Sun Pharmaceuticals, India) and 225 IU of highly purified human menopausal gonadotrophin (Menopur^®^ Ferring, Germany). Fourteen oocytes were retrieved - one at germinal vesicle (GV) stage and 13 - metaphase-II (M-II). Husband's ejaculated semen sample was allowed to liquefy and double layer density gradient (90 and 45%, Spermgrad^®^ Vitrolife, Sweden) was used for semen preparation. After 20 minutes of centrifugation at 300xg, pellet was aspirated and washed with sperm wash media. ICSI was performed on 13 M-II oocytes. The embryological details are summarized in Table 1. The woman failed to conceive in this cycle and a subsequent thaw embryo transfer cycle also failed.

**Table 1:** Outcome of intracytoplasmic injection of round headed spermatozoa in two cases

Case	Cycle no.	Eggs retrieved	Mature eggs	2PN (Fertilization rate%)	Embryos formed	Embryos transferred	Pregnancy	Outcome
1	I	14	13	4 (30.7)	4	2 (4/1,4/1)*	-	-
	Thaw	-	-	-	-	1(3/1)*	-	-
	II	7	7	2 (28.6)	2	2 (4/1,4/1)*	+	Abortion
2	I	15	10	2 (20)	1	1 (6/2)*	-	-
	II	13	8	1 (12.5)	1	1 (4/1)*	+	Singleton

* Represents cell/grade of embryo, e.g. 4/1 means 4 cell grade 1

A second fresh stimulation cycle was carried out as above with retrieval of seven M-II oocytes and transfer of two embryos. Serum β-hCG on day 15, after embryo transfer, was found to be positive. She had a spontaneous missed abortion at five weeks amenorrhea.

### Case 2

Another couple came to us with a history of primary infertility of nine years. The 29-year-old woman had a normal infertility workup with normal hysteroscopy, laparoscopy and hormonal profile. The 34-year-old male partner's previous three semen analyses showed five, 50 and 100% morphological abnormalities. A fresh semen sample at our clinic revealed a count of 35 million/ml, with 40% active motility and 100% having globozoospermia. The ovaries were stimulated using long protocol with 0.5 mg of Leuprolide acetate and 225 IU of recombinant FSH (Gonal-f^®^ Serono, Switzerland). Ten M-II and 5 GV stage oocytes were retrieved. Semen preparation was done as in case 1. A pregnancy did not ensue. A second ICSI attempt was made using the same stimulation protocol. The embryological details are summarized in Table 1. The subsequent embryo transfer resulted in a pregnancy which was uneventful. An elective caesarean section was done at 37 weeks with the birth of a healthy male child weighing 2.6 kgs.

## DISCUSSION

Only 21 pregnancies and 17 deliveries have been reported in globozoospermic patients (13 pregnancies and 10 deliveries without oocyte activation and rest with oocyte activation respectively).[11] Although ICSI procedure provides an answer to this problem of globozoospermia, a timely and detailed careful semen examination by appropriately trained and experienced personnel in such couples would save them from unnecessary investigations and treatment as seen in both the above cases.

The lack of acrosome reaction and failure to penetrate zona pellucida in IVF is overcome by the use of ICSI. But still the fertilization rates remain low ranging from zero to 100%.[11] We also observed a similarly low fertilization rate (12.5 - 30.7%).

Dam *et al*. found 99 cases of globozoospermia having been reported since the first Schirren case; no increase in abortion, miscarriage rates or aneuploidy/birth defects has been noted in the offspring of such men though the cases are few in number.[11]

The only treatment option in such cases is ICSI with or without oocyte activation. Repeated treatment cycles can be performed. No apparent predictors of success have been identified till now.
